# Extracellular miR-224 as a prognostic marker for clear cell renal cell carcinoma

**DOI:** 10.18632/oncotarget.22436

**Published:** 2017-11-15

**Authors:** Nakanori Fujii, Hiroshi Hirata, Koji Ueno, Junichi Mori, Shintaro Oka, Kosuke Shimizu, Yoshihisa Kawai, Ryo Inoue, Yoshiaki Yamamoto, Hiroaki Matsumoto, Tomoyuki Shimabukuro, Koichi Udoh, Yoshinobu Hoshii, Rajvir Dahiya, Hideyasu Matsuyama

**Affiliations:** ^1^ Department of Urology, Graduate School of Medicine, Yamaguchi University, Ube, Yamaguchi 755-8505, Japan; ^2^ Department of Urology, Ube Memorial Hospital, Ube, Yamaguchi 755-0051, Japan; ^3^ Center for Regenerative Medicine, Yamaguchi University Graduate School of Medicine, Ube, Yamaguchi 755-8505, Japan; ^4^ Department of Urology, Ube Kosan Central Hospital, Ube, Yamaguchi 755-0151, Japan; ^5^ Institute for Biomedical Research and Education, Yamaguchi University Science Research Center, Ube, Yamaguchi 755-8505, Japan; ^6^ Department of Diagnostic Pathology, Yamaguchi University Hospital, Ube, Yamaguchi, 755-8505, Japan; ^7^ Department of Urology, San Francisco Veterans Affairs Medical Center and University of California, San Francisco, California 94121, USA

**Keywords:** microRNA, miR-224, exosome, clear cell renal cell carcinoma

## Abstract

Exosome-miRNAs (exo-miR) have recently been identified as modulators of cancer progression and distant metastasis. We previously found that intracellular miR-224 is up-regulated and significantly related to cancer invasion and metastasis in clear cell renal cell carcinoma (ccRCC). We therefore investigated the role of exosome miR-224 in ccRCC and explored the interaction between intra- and extracellular miR-224 in renal cell carcinoma. To validate the method for isolating exosomes from blood samples or cell culture media, we examined exosome morphology using transmission electron microscope (TEM). We investigated the relationship between exo-miR-224 expression and patient prognosis in 108 ccRCC patients. We isolated exosomes from a metastatic renal cancer cell line and tested their effects on a primary renal cancer cell line with several functional analyses. We found that the high expression level exo-miR-224 group has significantly shorter progression-free survival, cancer-specific survival, and overall survival compared with the low expression group. In multivariate analysis, a high level of exo-miR-224 was a significant risk factor related to all prognoses investigated. After adding exosomes from a metastatic RCC cell line to a primary RCC cell line, cell proliferation and invasion were increased while the percentage of apoptotic cells was significantly decreased. Intracellular levels of miR-224 were significantly up-regulated in the primary renal cancer cell line. Extracellular miR-224 in exosomes impacts on patient prognosis and is a potential prognostic biomarker for ccRCC patients.

## INTRODUCTION

Renal cell carcinoma (RCC) accounts for 2% of all cancer diagnoses and cancer deaths worldwide [1]. Approximately 75% of RCCs are clear cell RCCs (ccRCCs), and ccRCC is the major cause of cancer-specific mortality (CSM) [2, 3]. Localized RCC can be treated with partial or radical nephrectomy. However, ccRCC is regarded as resistant to chemotherapy and radiotherapy [4, 5] and 20%–30% of localized RCC patients experience local or distant recurrence [6]. Several targeted drugs can be used to treat advanced RCC. The treatment response is varied and most patients develop to progression and finally die from the cancer [7]. Although several prognostic models of RCC based on clinico-pathological parameters have been proposed, they lack accuracy [5, 8]. To obtain better treatment results, early prediction or identification of patients with aggressive disease or poor prognosis is needed, but, compared with other cancers, there are very few tumor markers for ccRCC and no established prediction marker [9, 10]. Hence, identification of novel tumor prediction markers is greatly needed and may provide new therapeutic approaches for ccRCC patients.

MicroRNAs (miRNAs) are small, non-coding RNAs, ∼22 nucleotides in length that are capable of regulating gene expression at transcriptional and translational levels [11]. A number of studies have investigated intracellular miRNAs in several cancers [12]. miRNAs can repress translation of mRNA to protein via binding to the 3′-UTR of a target mRNA, resulting in regulation of target gene expression [13].

Liquid biopsies (e.g. urine, blood, saliva) are non-invasive and contain clinically relevant biomarkers to detect premalignant and early stage cancers [14, 15]. Liquid biopsies contain exosomes, 20–150 nm-sized vesicles that are released from many kinds of cells, including cancer cells, into the extracellular space [16, 17]. Recently miRNAs have been identified in exosomes, and function as key players in cancer invasion and metastasis [18, 19]. Extracellular miRNAs may play roles as mediators of cell-to-cell interaction or communication and can serve as biomarkers.

Several miRNAs were reported to be overexpressed in RCC [20, 21]. We recently found that miR-224 was significantly overexpressed in ccRCC tissues compared with normal kidney tissues. Moreover, miR-224 is highly expressed in ccRCC tissues, compared with normal kidney, according to the Cancer Genome Atlas (TCGA) database and work by Li *et al*. [21].

In this study we investigated the role of exosome miR-224 as a new biomarker. We also performed functional analysis of exosome-miRNA-224 and intracellular miRNA-224. We also investigated the interaction between intracellular miRNA and exosomal miRNA using primary and metastatic renal cancer cell lines. To our knowledge, this is the first report to show that extracellular miR-224 may function as a mediator of cell-to-cell interaction or communication in regard to cancer invasion and metastasis in RCC and that miR-224 may serve as a new biomarker for RCC.

## RESULTS

### miRNA-224 expression in ccRCC tissues and cell lines

We performed quantitative real time RT-PCR to clarify whether miR-224 was upregulated in human ccRCC tissues. miR-224 expression was significantly higher in ccRCC tissues (*n* = 20) compared with matched normal kidney tissues (*n* = 20) (Supplementary Figure 1). miR-224 expression was also higher in renal cancer cell lines compared with a normal kidney cell line (RPTEC) (Supplementary Figure 1).

### Effect of upregulation of miR-224 on the 769-P RCC cell line and the RPTEC human renal proximal tubule cells

**Figure 1:**
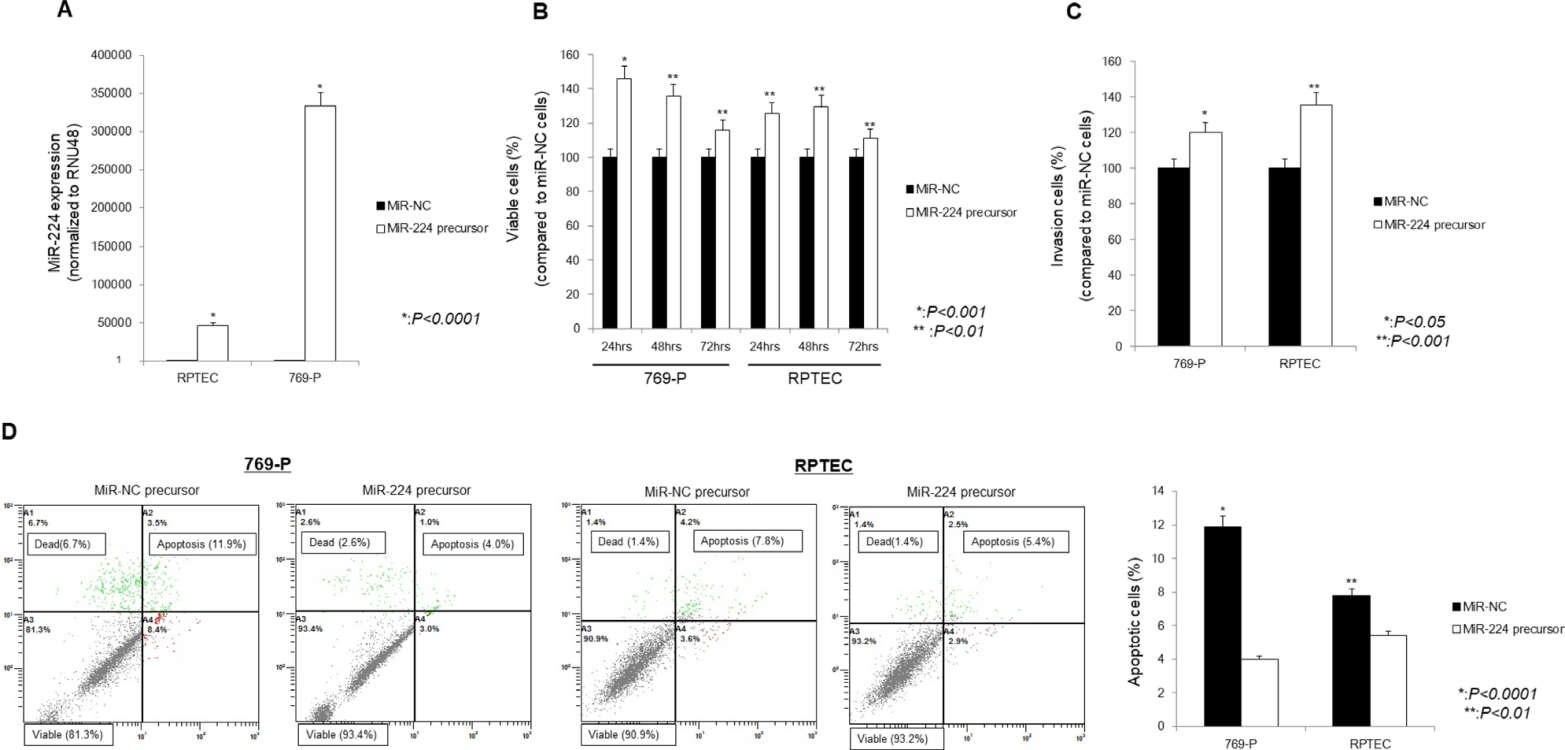
Effect of miR-224 upregulation on 769-P cells and RPTEC cells (**A**) qRT-PCR. In 769-P cells and RPTEC cells transfected using an miR-224 precursor, miR-224 expression was significantly increased compared with that in cells transfected by a miR-NC precursor. (**B**) MTS assay. Cell viability was significantly increased at 24 h, 48 h, and 72 h in cells transfected with the miR-224 precursor compared with control cells. (**C**) Invasion assay. The number of invading cells significantly increased in cells transfected 769-P and RPTEC. (**D**) Apoptosis assay. The percentage of apoptotic cells significantly decreased in 769-P and RPTEC cells transfected with the miR-224 precursor compared with control cells.

After up-regulation of miR-224 in the 769-P RCC cell line and the RPTEC normal kidney cell line using an miR-224 precursor (Figure 1A), cell viability and invasion ability were significantly increased, whereas the number of apoptotic cells was significantly decreased compared with control cells (Figure 1B–1D).

### Effect of downregulation of miR-224 on Caki-1 and Caki-2 RCC cell lines

After down-regulation of miR-224 in RCC cell lines (Caki-1 and Caki-2), using an miR-224 inhibitor, cell viability and invasion ability were significantly decreased whereas the number of apoptotic cells was significantly increased compared with control cells (Supplementary Figure 2).

### Exosomes in human serum and cell culture media

**Figure 2:**
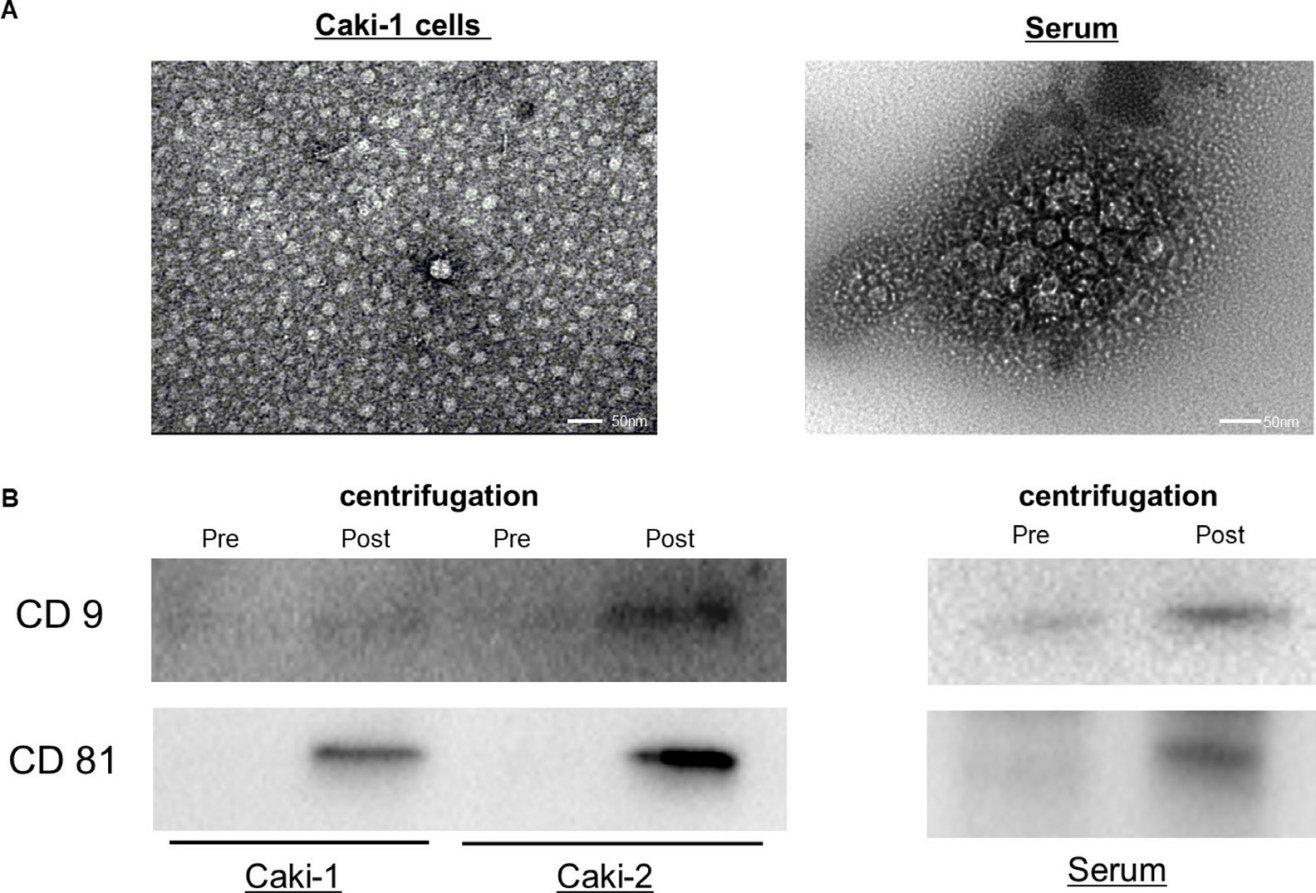
Exosomes from human serum and cell culture medium (**A**) Exosomes extracted from Caki-1 cell culture medium and serum were observed using transmission electron microscopy. (**B**) Western blots showed the expression of CD9 and CD81. The CD9 and CD81 bands were more intense in exosomes after ultracentrifugation compared with those before ultracentrifugation.

Transmission electron microscopy analysis of human serum and cell culture media without FBS revealed rounded membrane-bound vesicles under 200 nm in size (Figure 2A) that expressed CD9 and CD81on their surface (Figure 2B).

### Relationship between exo-miR-224 expression level and RCC patient prognosis

**Figure 3:**
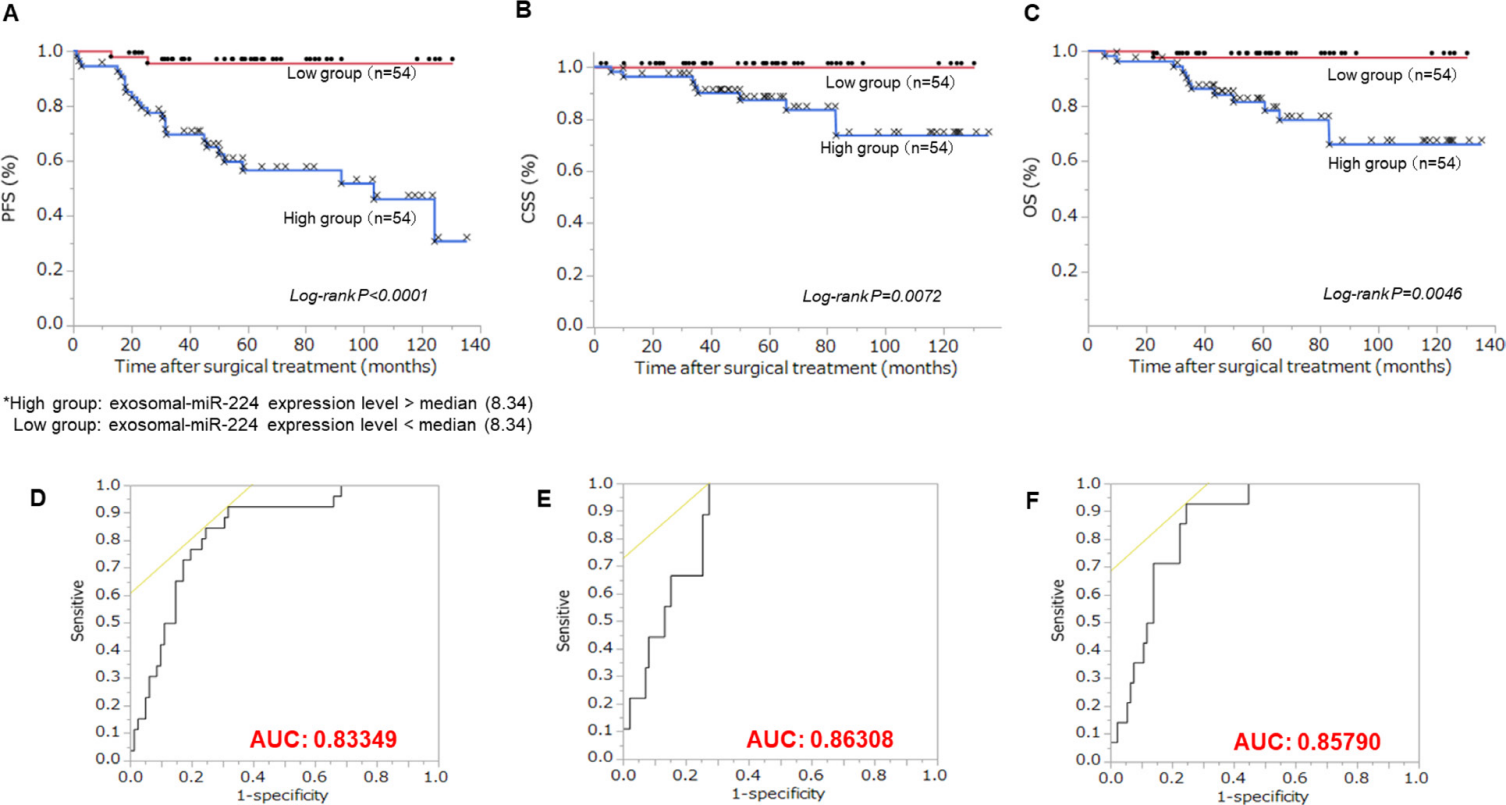
Relationship between extracellular miR-224 expression and prognosis Patients were divided to two groups of 54 according to median extracellular miR-224 expression. (**A**) Kaplan-Meier plot of progression-free survival (PFS). High exo-miR-224 group had significantly worse PFS than the low exo-miR-224 group (Log-rank *P* < 0.0001). (**B**) Kaplan-Meier plot of cancer-specific survival (CSS). High exo-miR-224 group had significantly worse CSS than the low exo-miR-224 group (Log-rank *P* = 0.0072). (**C**) Kaplan-Meier plot of overall survival (OS). High exo-mi-224 group had significantly worse PFS than the low exo-miR-224 group (Log-rank *P* = 0.0046). (**D**) ROC curve of progression using extracellular miR-224 (AUC: 0.833). Extracellular miR-224 was a good marker for progression. (**E**) ROC curve of cancer-specific mortality using extracellular miR-224 (AUC: 0.863). Extracellular miR-224 was good marker for cancer specific mortality. (**F**) ROC curve of overall survival using extracellular miR-224 (AUC: 0.857). Extracellular miR-224 was good marker for overall survival.

We divided RCC patients into two groups based on median exosomal miR-224 expression level. The high expression level exosomal miR-224 group had significantly shorter progression-free survival (PFS), cancer-specific survival (CSS), and overall survival (OS) compared with the low level expression group (Figure 3A–3C, log-rank *P* < 0.0001, log-rank *P* = 0.0072, log-rank *P* = 0.0046, respectively). ROC curves and AUC are shown Figure 3D–3F Moreover, we evaluated the prognostic significance of clinico-pathological parameters, including gender, age, stage, Fuhrman grade, lympho-vascular invasion and exo-miR-224 expression level in ccRCC patients (Table 1). High exosomal miR-224 expression was a significant independent risk factor related to PFS, CSS, and OS in multivariate analysis (HR = 11.0; *P* < 0.0001, HR = 1.6; *P* = 0.0140, HR = 9.1; *P* = 0.0043, respectively).

**Table 1:** Univariate and Cox proportional hazards regression analysis of factors related to progression, cancer-specific mortality, and overall survival

	Progression				Cancer-specific mortality				Overall survival			
	Univariate		Multivariate		Univariate		Multivariate		Univariate		Multivariate	
Parameters	HR (95%CI)	P-value	HR (95%CI)	P-value	HR (95%CI)	P-value	HR (95%CI)	P-value	HR (95%CI)	P-value	HR (95%CI)	P-value
Gender												
Male	1				1				1			
Female	1.0 (0.8–1.2)	0.8177			1.0 (0.9–1.1)	1.0000			0.9 (0.8–1.1)	0.5608		
Age												
<65 y.o	1				1				1			
≥65 y.o	1.0 (0.8–1.2)	1.0000			1.0 (0.9–1.1)	1.0000			1.0 (0.9–1.2)	0.7755		
Stage												
I	1		1		1		1		1		1	
≥II	1.3 (1.0–1.8)	0.0164	3.0 (1.3–7.1)	0.0123	1.2 (1.0–1.4)	0.0267	1.8 (1.1–2.8)	0.0114	1.2 (1.0–1.4)	0.0428	3.0 (1.1–9.2)	0.0424
Fuhrman grade												
G1/2	1		1		1				1			
G3/4	1.4 (1.0–1.9)	0.0134	1.8 (0.8–4.3)	0.1481	1.1 (1.0–1.3)	0.0752			1.2 (0.9–1.4)	0.1134		
LVI												
Yes	1				1				1			
No	1.7 (1.0–2.7)	0.0568			1.9 (1.1–3.2)	0.0856			1.4 (0.7–2.5)	0.3855		
Exosomal-miR-224												
Low	1		1		1		1		1		1	
High	1.7 (1.4–2.2)	<0.0001	11 (3.3–68.7)	<0.0001	1.2 (1.1–1.4)	0.0027	1.6 (1.1–2.5)	0.0140	1.3 (1.1–1.5)	0.0009	9.1 (1.8–166.1)	0.0043

HR, hazard ratio; CI, confidence interval; LVI, lymphovascular invasion.

### The relationship between intracellular and extracellular miR-224 in cell lines

**Figure 4:**
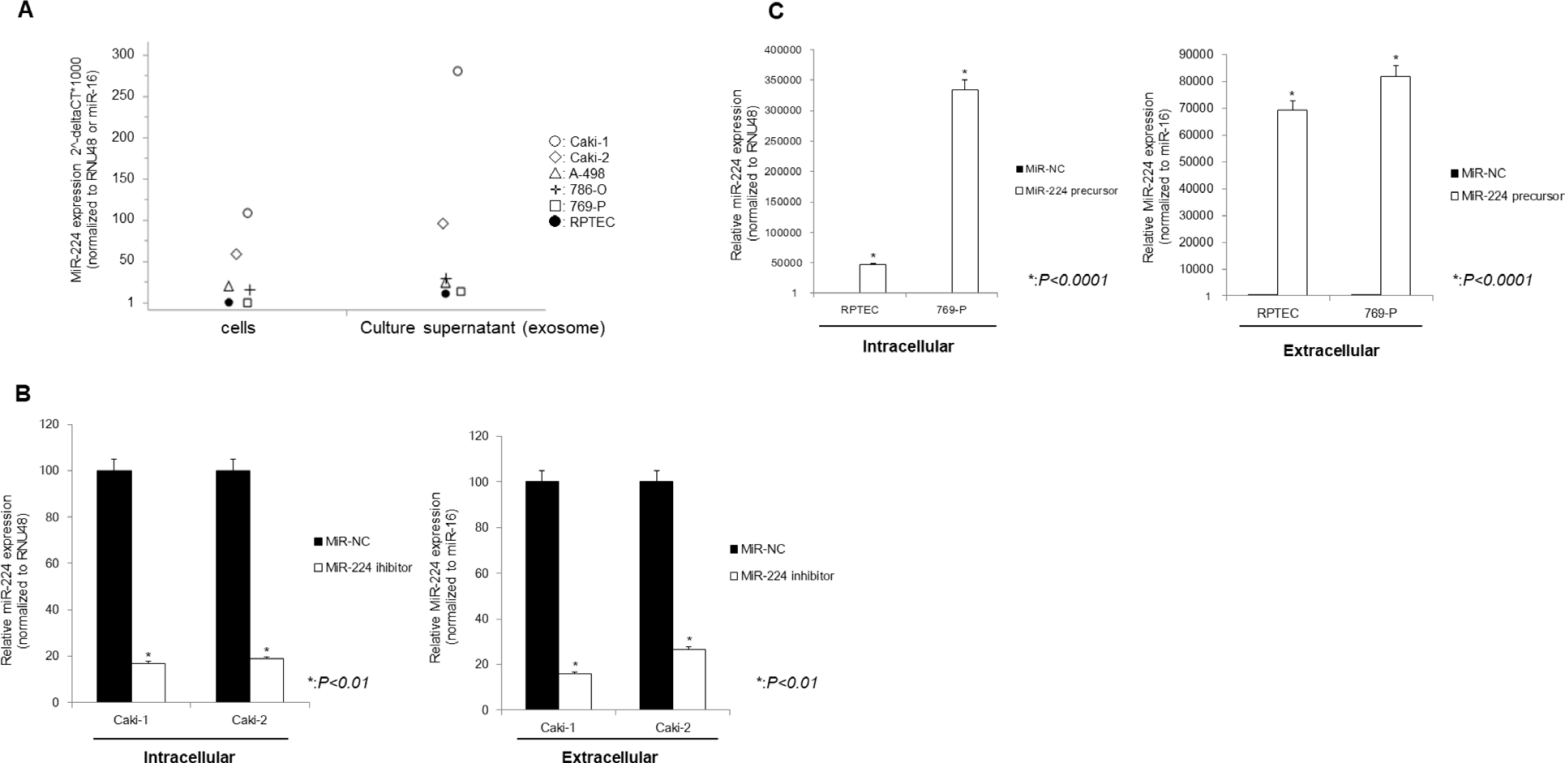
Relationship between intracellular and extracellular miRNA-224 in cell lines (**A**) The expression of exo-miR-224 paralleled that of intracellular miRNA-224. (**B**) After knock-down of intracellular miR-224, extracellular levels of miR-224 were also significantly decreased. (**C**) After overexpression of intracellular miR-224, extracellular levels of miR-224 were also significantly increased.

Among renal cell cancer cell lines, Caki-1 and Caki-2 had high and 769-P and RPTEC had low expression levels of miR-224 (Figure 4A). The level of exosomal miR-224 expression was also high in Caki-1 and Caki-2 cells and low in 769-P cells and RPTEC. We, therefore, selected Caki-1, Caki-2, 769-P and RPTEC cells for further functional analysis. After knock-down of intracellular miR-224 in Caki-1 cells using an miR-224 inhibitor, extracellular levels of miR-224 were also significantly decreased (Figure 4B). In contrast, extracellular levels of miR-224 were significantly increased after intracellular miRNA-224 overexpression in RCC cells (Figure 4C).

### Cell to cell interaction of exosomes and microRNA

**Figure 5:**
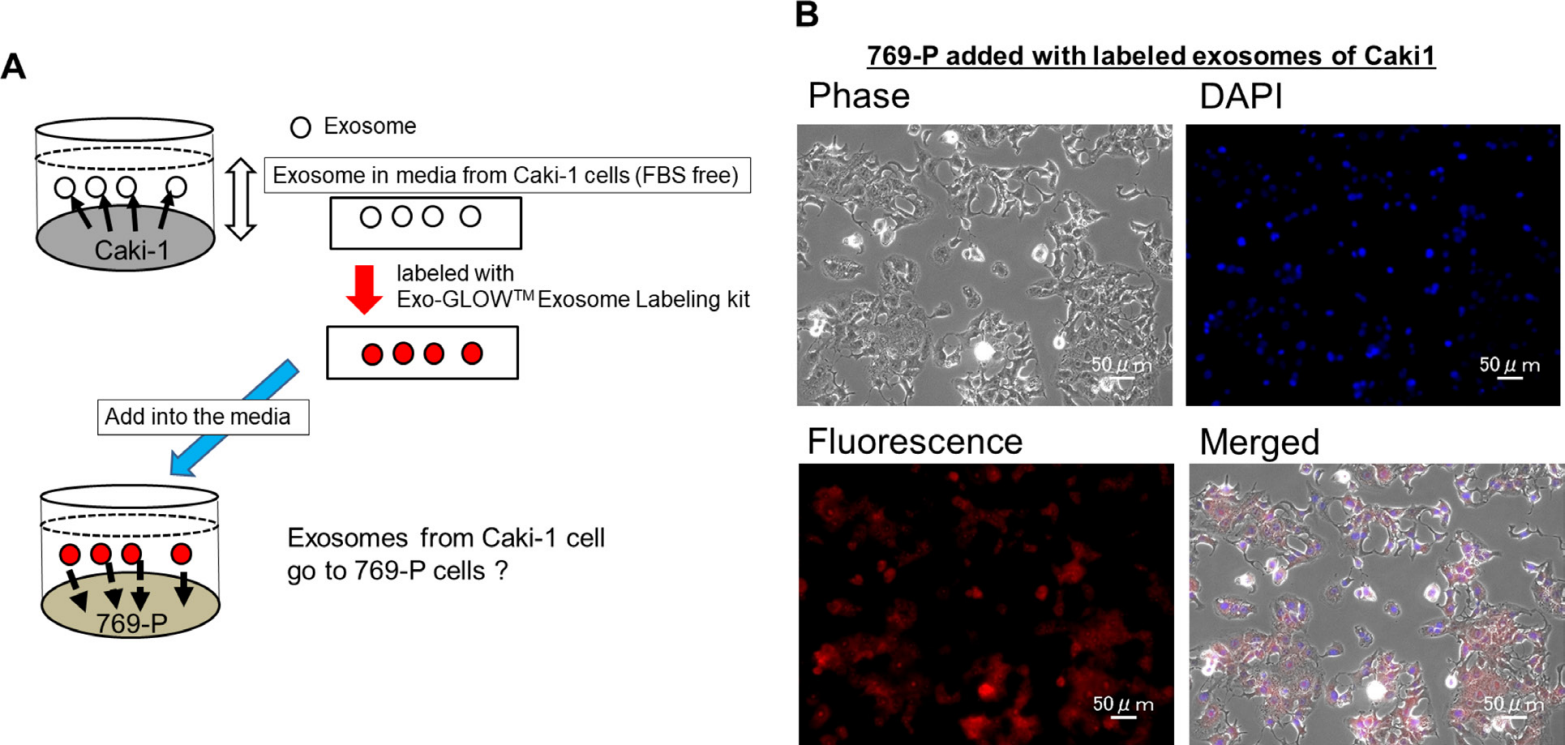
Cell to cell interaction of exosomes (**A**) Scheme of the experiment. (**B**) Caki-1 exosomes were labeled using an Exo-GLOWTM Exosomes labeling kit. Immunofluorescence staining showed that labeled exosomes were incorporated into 769-P cells.

The experimental strategy is shown in Figure 5A. Initially we collected exosomes from the cell culture medium of Caki-1 cells. Then the exosomes released from Caki-1 cells were labeled using an Exo-GLOW Exosome Labeling kit (System Biosciences, Palo Alto, CA, USA). The stained exosomes were added to 769-P cell medium. Two hours after the addition of exosomes, we observed labeled exosomes incorporated into 769-P cells using fluorescence microscopy (Figure 5B).

**Figure 6:**
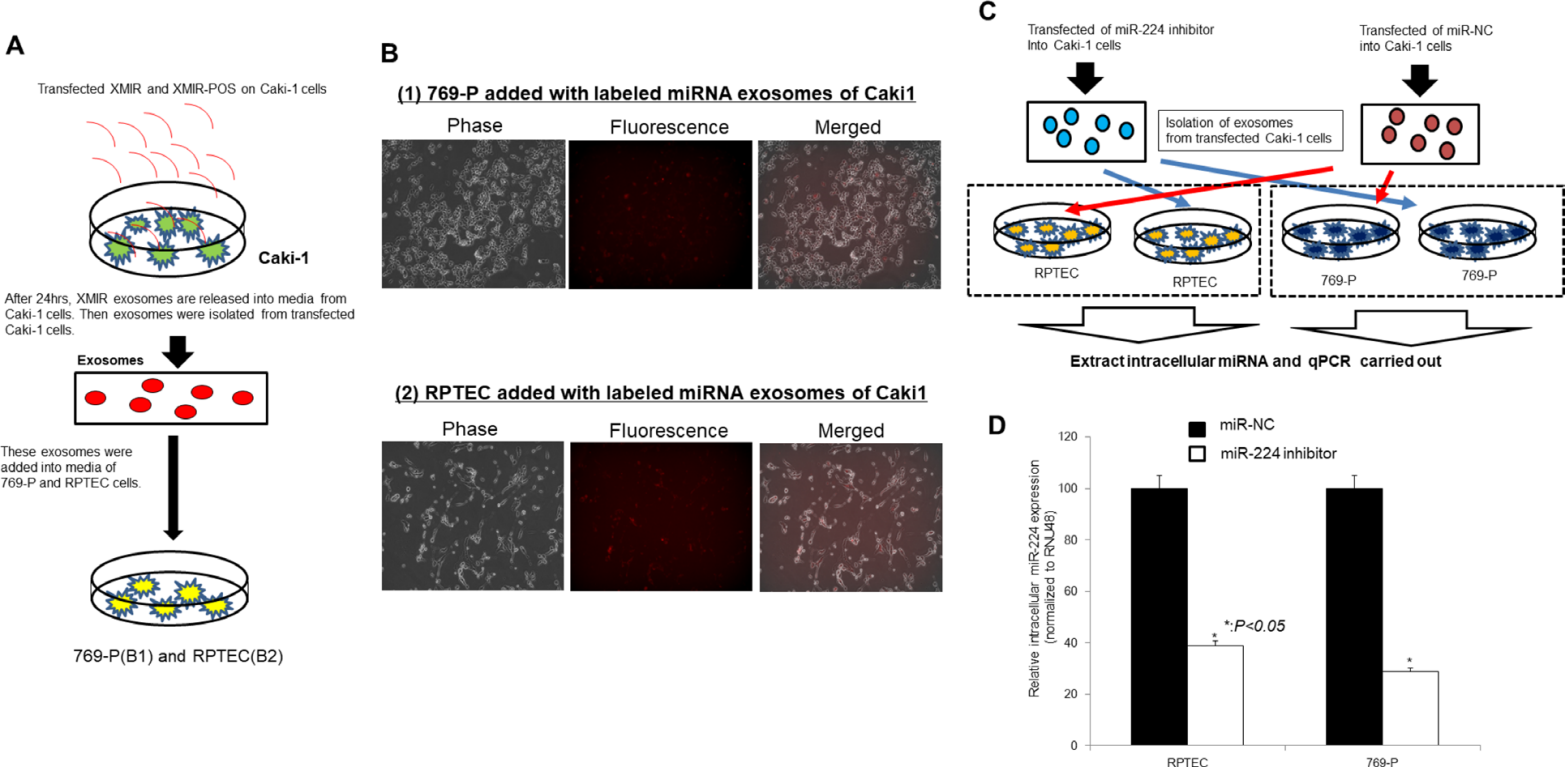
Exosome miRNA from cells to cells experiment (**A**) Schema of the labeling exosome miRNA-oligo experiment. (**B**) Validation of target cell delivery (Caki-1 to 769-P/RPTEC cells) using positive control using fluorescence microscope. Cell (769-P and RPTEC) imaging on fluorescence microscope using a standard RFP filter set to visualize the Texas-Red. signal conjugated to the XMIR-positive control oligo. (**C**) Schema: exosomes of Caki-1 transfected miR-224 inhibitor or miR-NC was added to 769-P cells and RPTEC cells. (**D**) Intracellular miR-224 expression in 769-P and RPTEC depending on extracellular miR-224 expression in Caki-1 cells

We also carried out labeled miRNA experiment using XMIR RNA Oligo Kits (System Biosciences). As shown in Figure 6A, labeled microRNA oligo were transfected to Caki-1 cells. The purchased oligo is designed to fuse the XMotif sequence to a miRNA which results in exosomal loading of the RNA oligo. So the oligo is first transfected into a culture of the exosome generating cell type such as Caki-1 cell. After 24 hours, exosomes packed with the XMIR in cell culture media are precipitated. Then the exosomes were added to target cells such as 769-P and RPTEC. As shown in Figure 6B, the target cells such as 769-P and RPTEC are visualized on a fluorescence microscope, showing that initial oligos in Caki-1 cells are included in target cells (769-P and RPTEC). Under the similar condition, we next knocked down of miR-224 in Caki-1 cells and then exosomes are collected and the exosomes are added to the culture media of target cells such as 769-P and RPTEC (Figure 6C). The expression of intracellular miRNA-224 in target cells was paralleled with those in initial Caki-1 cells (Figure 6D).

**Figure 7:**
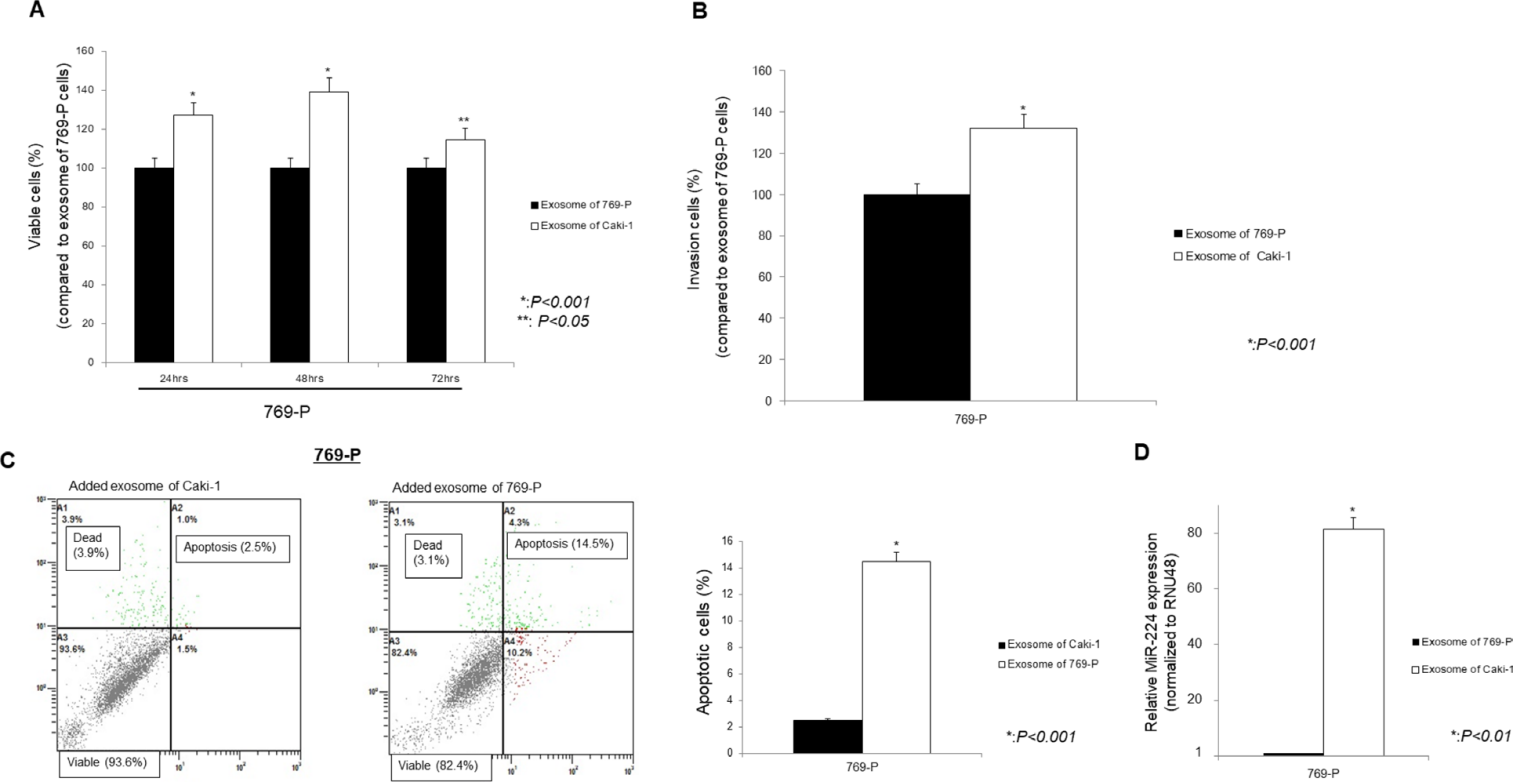
Effect of addition of Caki-1 exosomes to target 769-P cells using the MTS assay (**A**), Cell Invasion assay (**B**), Apoptosis assay (**C**), and on the expression of miR-224 (**D**). (A–C) Caki-1 exosomes added to 769-P cells significantly promoted cell viability and cell invasion, whereas the number of apoptotic cells was significantly decreased compared with 769-P exosomes added to 769-P cells. (D) Intracellular miRNA-224 expression was significantly increased after addition of Caki-1 exosomes to 769-P cells.

Under similar conditions, functional analyses were performed. The miR-224 expression was significantly increased and cell viability and invasion ability were significantly promoted, while the number of apoptotic cells was significantly suppressed in 769-P cells added exosomes of Caki-1 compared with 769-P cells added exosomes of 769-P (Figure 7A–7C).

RPTEC cells added exosomes of Caki-1 were significantly increased the ability to cell viability and invasion compared to RPTEC cells added exosomes of RPTEC (Supplementary Figure 3)

## DISCUSSION

Blood samples can contain several tumor components, including cell free tumor DNA, circulating tumor cells, and exosomes [22]. Recent studies have shown that various cells release exosomes into urine and blood. Exosomes contain several molecules, such as DNA, proteins, and miRNAs. The miRNAs in blood exosomes are protected from endogenous RNase activity [23]. miRNAs are emerging as important regulators of tumor metastasis because miRNAs modulate gene expression at the post-transcriptional level through multifaceted suppression of mRNA targets with complimentary sequences [24]. Exosomes influence every step of cancer metastasis and can be targeted by oncological treatment [25]. Thus, among molecules in exosomes, miRNAs have been investigated due to their regulatory role in gene expression [26]. Some reports have shown that oncogenic miRNA are associated with prognosis or as useful biomarkers in ccRCC [20, 27, 28]. Among oncogenic miRNAs, miR-224 is upregulated and plays an oncogenic function in hepatocellular carcinoma [27, 29, 30], esophageal squamous cell carcinoma [31], colorectal cancer [32, 33], and non-small cell lung cancer [34]. Thus miR-224 is regarded as an oncogenic miRNA in several cancers. miR-224 was also reported to be overexpressed in ccRCC by TCGA database and some reports [21, 35]. Linchner *et al.* reported that *VHL*, a crucial tumor suppressor gene in RCC, is a direct target of miR-224 [36]. However, the actual function of miR-224 associated with ccRCC tumor growth or progression remains unknown. In this study, our data also showed that miR-224 expression was significantly higher in ccRCC tissues compared with that in adjacent normal kidney tissues.

If the expression of an miRNA is high in cancer tissues, it is generally considered to be oncogenic. However, in prostate cancer, miR-224 is highly expressed in tissues associated with perineural invasion [37], but functional analyses in prostate cancer revealed a tumor suppressor function of miR-224 [38, 39]. Therefore, to understand the function of miR-224 in RCC, functional analysis is very important. Our *in vitro* functional analyses, showed that miR-224 promoted cell viability and invasion, and suppressed apoptosis. Therefore, miR-224 may have an oncogenic function related to tumor growth or progression in ccRCC. Several previous reports showed similar results for miR-224 in other cancers [27–32, 36, 40]. miR-224 may be considered as a biomarker for prognosis and high miR-224 expression group patients may be candidates for careful follow-up after surgery.

Exosomal miRNAs in the blood are of increasing interest for their potential as new cancer biomarkers because exosomal miRNAs are protected from degradation. In some cancers, serum exosomal microRNAs were significantly up-regulated in hepatocellular carcinoma compared with chronic hepatitis B and liver cirrhosis [41]. Sohn *et al*. concluded serum exosomal microRNAs may be a novel serological biomarker for HCC [41].

One of the aims of this study was to identify new exosomal miRNAs as non-invasive biomarkers to detect premalignant and early stage ccRCC. We found that intracellular miRNA expression is significantly higher in ccRCC tissues compared with normal kidney tissues. Rabinowits *et al.* have shown a similarity between patterns of circulating exosomal miRNAs and tumor-derived miRNAs in patients with adenocarcinoma of the lung [42]. Even though miRNA levels are high in host cancer cells, it was unknown whether the miRNA levels in exosomes derived from host cells was high or not. Thus, we investigated the correlation between miR-224 expression in ccRCC tissues (intracellular miRNA) and serum (extracellular miRNA) from the same patients. We found a positive correlation for miR-224 levels between ccRCC tissues and serum (data not shown), suggesting that the miR-224 level in exosomes derived from ccRCC cells may reflect the expression of miR-224 in host ccRCC tissues. Next, we found that exosomal miR-224 expression was associated with poor prognosis using the Kaplan-Meier survival curve. Moreover, the Cox proportional hazards model showed that high exo-miR-224 expression was an independent risk factor for patient prognosis. Our study is the first to show that exosomal miR-224 levels parallel those of miRNA-224 in ccRCC tissues and are significantly associated with prognosis in ccRCC patients. Interestingly Petrozza *et al.* have reported that miR-210-3p extracted from urinary specimens in surgical treatment is useful as a non-invasive biomarker [28]. Moreover, expression of miR-210-3p extracted from urinary specimens in surgical treatment was associated with complete surgical resection or response to treatment in ccRCC [28]. Another report showed miR-210 was potential biomarker for ccRCC diagnosis [43]. Exosomes were non-invasive biomarker as well as urinary specimen. Exosomal miRNA may detect more aggressive and poorer prognosis ccRCC patients using non-invasive methods. Exo-miRNA in blood or urine may be a non-invasive and promising biomarker to detect micro-invasion or cancer metastasis after surgery in ccRCC patients.

After knocking down miR-224 in renal cancer cell lines, extracellular miRNA-224 levels were also significantly decreased. In contrast, when miR-224 was overexpressed in cells by miR-224 precursor transfection, extracellular miR-224 was significantly increased. This supports the idea that exosomal miRNA-224 expression reflects intracellular miRNA-224 levels in renal cancer cells.

Our next aim was to investigate exosome function in renal cancer invasion and metastasis via *in vitro* interaction between intracellular and extracellular miRNAs. We collected exosomes from cell culture media derived from the metastatic RCC cell line, Caki-1. We fluorescently stained these exosomes and added them to the medium of the primary RCC cell line, 769-P and normal kidney cell line, RPTEC. Using fluorescence microscopy, we found that the 769-P cells and RPTEC cells were stained, indicating in the uptake of Caki-1 exosomes by 769-P cells and RPTEC cells. Under these conditions, cell proliferation and invasion abilities were significantly increased, suggesting that exosomes derived from Caki-1 cells induced malignant features in 769-P cells. Cell invasion is a very important feature of metastasis and several studies have shown the importance of exosomes in tumor metastasis [25, 44, 45]. Some investigators have reported that exosomes isolated from metastatic cells increased the metastatic potential of primary cancers [46, 47]. Thus, exosomes play a role in the establishment of the metastatic niche by communication between host cancer cells and recipient cells. Moreover, our data indicate that exosomes derived from metastatic renal cancer cells associated with cancer progression of primary renal cancer cells, which is consistent with previous reports [46, 47]. We also found oncogenic intracellular miRNA-224 levels were significantly increased after exosome treatment. These results suggest that extracellular miRNA-224 is a mediator of cell-to-cell communication that can transmit information from an established metastatic site to occult micro-metastases.

In conclusion, we show that extracellular miR-224 can serve as a new biomarker and may mediate cell-to-cell interaction or communication in regard to cancer invasion and metastasis in RCC. miR-224 itself has an oncogenic function related to tumor growth and progression.

## MATERIALS AND METHODS

### Clinical samples

In total, 108 patients who underwent partial or radical nephrectomy at Yamaguchi University Hospital from October 2005 to December 2014 were enrolled in this study. All patients were pathologically diagnosed with ccRCC and patients with lymph node or distant metastasis were excluded from the study. Blood sampling was performed before surgery. Detailed patient characteristics are shown in Table 2. Our study was approved by the institutional ethics committees of the Graduate School of Medicine, Yamaguchi University and written informed consent was obtained from all individuals enrolled in the study.

**Table 2:** Characteristic of 108 patients who underwent partial or radical nephrectomy

Variables	Cases	%
Gender		
Male	67	62.0
Female	41	38.0
Age (years)		
Median	64.5	
Range	35–96	
T stage (pathological)		
1a	51	47.2
1b	20	18.5
2	10	9.3
3a	20	18.5
3b	6	5.6
3c	1	0.9
Fuhrman grade		
G1/2	76	70.4
G3/4	32	29.6
Follow up duration (months)		
Median	54.9	
Range	2.3–135.4	
Progression		
No	82	75.9
Yes	26	24.1
Cancer-specific death		
No	99	91.7
Yes	9	8.3
Overall survival		
Survival	94	87.0
Death	14	13.0

LVI; lymphovascular invasion.

### RCC and normal cell lines

Primary renal cancer cell lines (769-P; ATCC number: CRL-1933, Caki-2; ATCC number: HTB-47) and metastatic renal cancer cell line (Caki-1; ATCC number HTB-46) were purchased from the American Type Culture Collection (Manassas, VA, USA).

769-P was cultured in RPMI 1640 medium (Life Technologies, Carlsbad, CA, USA) supplemented with 10% fetal bovine serum (FBS) and maintained in humidified incubators (5% CO_2_). Caki-1, and Caki-2 were cultured in McCoy's 5A medium (Life Technologies) supplemented with 10% FBS and maintained in humidified incubators (5% CO_2_)

Human renal proximal tubule cells (RPTEC; catalog #CC-2553) were from Lonza (Basel, Switzerland). Renal epithelial growth media (catalog #CC-3191 and CC-4127) from Lonza was used for RPTEC cells.

### Isolation of exosomes from serum or cell culture media

Exosomes were isolated from serum using a Total Exosome Isolation kit (from serum) (Invitrogen, Waltham, Massachusetts, USA). Briefly, serum was obtained by centrifugation at 2000 × g for 30 minutes to remove cells and debris. Then the supernatant containing the clarified serum was transferred to new centrifuge tubes and mixed with Total Exosome Isolation reagent. After incubation at 4°C for 30 minutes, the sample was centrifuged at 10,000 × g for 10 minutes. Exosomes were contained in the pellet. Exosomes were also isolated from cell culture media using the Total Exosome Isolation kit (from media). Usually fetal bovine serum includes exosomes. Thus we exchanged the regular cell culture media to FBS-free media 24 hours before exosome isolation.

### Transmission electron microscopy (TEM)

A carbon-coated grid was put on a sheet of Parafilm. The samples (exosome or control) were placed on the grid for 10 minutes and excess liquid was drained. Then 2% uranyl acetate was added for 10 seconds and excess liquid was drained. The grid was kept for 10 minutes until dry. We examined the morphology of exosomes using a transmission electron microscope (Tecnai, FEI, Hillsboro, Oregon, USA).

### Exosome immunoprecipitation and western analysis

Exosome Immunoprecipitation Reagent (Protein A) is designed for immunoprecipitation of exosome protein (Exosome Immunoprecipitation, Thermo Fisher Scientific, Waltham, MA, USA). Briefly, an anti-CD9 antibody and anti CD81 antibody (an exosome surface marker), was added to Dynabeads coated with Protein A and then incubated with rotation for 10 minutes at room temperature. The samples were then placed on magnetic stands and the supernatant was removed. After washing the magnetic bead complexes (Dynabeads-ProteinA-anti-CD9 antibody and Dynabeads-ProteinA-anti-CD81 antibody), exosome samples were added to the beads. Exosomes in serum or culture media can be immunopurified directly. The beads-antigen-antibody complex was incubated with rotation for 10 minutes and then washed several times with PBS. After washing, the Dynabeads-antibody-antigen complexes were re-suspended. Western analysis was performed using an anti-CD9 antibody and a secondary antibody.

### miRNA isolation from exosomes (serum and cell culture media)

miRNAs were isolated from exosomes using a Total Exosome RNA and Protein Isolation kit (Life Technologies, Carlsbad, CA, USA) according to the manufacturer's instructions.

### miRNA inhibitor and miRNA precursor transfection

Anti-miR™ miRNA inhibitors [negative control (miR-NC inhibitor) or miR-224 inhibitor (miR-224 inhibitor), Ambion, Foster City, CA, USA] were transiently transfected into cells using Lipofectamine RNAiMAX (Invitrogen), according to the manufacturer's instructions. Pre-miR™ miRNA precursors [negative control (miR-NC) or hsa-miR-224 (miR-224), Ambion] were transfected into cells using Lipofectamine RNAiMAX, according to the manufacturer's instructions. After transfection, cells were incubated at 37°C for 48 h until further treatment.

### Total RNA extraction from tissues and cells

RNA (miRNA, and total RNA) was extracted from formalin-fixed, paraffin-embedded (FFPE) human renal cancer and adjacent non-cancerous normal kidney tissues using an miRNeasy FFPE kit (Qiagen, Hilden, Germany) after micro-dissection based on a pathologist's review. Total RNA was also extracted from frozen tissues and renal cancer cell lines using a miRNeasy mini kit (Qiagen), according to the manufacturer's protocol. DNA was digested with a Qiagen RNase-Free DNase kit (#79254), according to the manufacturer's protocol.

### Quantitative real-time RT-PCR

Quantitative real-time RT-PCR was performed in triplicate with an Applied Biosystems StepOnePlus using TaqMan universal PCR master mix according to the manufacturer's protocol (Applied Biosystems, Foster City, CA, USA). The TaqMan probes and primers were purchased from Applied Biosystems. Human RNU48 (Assay ID: 001006) and miR-16 (Assay ID: 000391) were used as endogenous controls. Levels of miR-224 (Assay ID: 000599) RNA expression were determined using StepOnePlus software (Applied Biosystems). The miRNA expression levels were determined using the 2^^-deltaCt^ method.

### Cell to cell exosome and microRNA experiments

Exosomes in the culture medium of Caki-1 cells were isolated and fluorescently stained using an Exo-Glow Exosome Labeling Kit (System Biosciences, Palo Alto, CA, USA). The same fluorescently stained exosomes added into 769-P cell media. After 2 hours incubation, We confirmed the exosomes intake in 769-P cells by fluorescent microscopy.

We also labeled miRNA oligos in the culture medium of Caki-1 cells using XMIR-POS (System Biosciences, Palo Alto, CA, USA). After 24 hours, exosomes were isolated from transfected Caki-1 cells culture supernatant. Fluorescently stained exosomes were added to the culture media of 769-P cells and RPTEC cells. We examined whether fluorescently stained exosomes could move to other cells using a fluorescence microscope (BZ-X700, Keyence Corporation, Osaka, Japan).

### Cell viability, cell invasion, and apoptosis assays

After the addition of Caki-1 exosomes to 769-P media, we performed functional analyses. Cell viability was tested using the MTS assay (CellTiter 96 Aqueous One Solution Cell Proliferation Assay, Promega). Cell viability was measured at 24, 48, and 72 h after cells were plated at OD 490 nm. Data are the mean ± S.D. of three independent experiments. Cell invasion assays were performed using the CytoSelect 24-well cell invasion assay kit (Cell BioLab, San Diego, CA, USA). Transfected cells were transferred to the upper chamber in triplicate. After 48 hours incubation at 37°C (5% CO_2_), cells migrating through the membrane were stained and the results are expressed as migrated cells quantified at OD 560 nm after extraction, according to the manufacturer's instructions.

The apoptotic distribution of cells in each sample was determined using FACS analysis (Cytomics FC500, Beckman Coulter, Fullerton, CA, USA) accordingly our previous report [48]. Data are the mean ± S.D. of four independent experiments.

### Statistical analysis

Categorical variables were compared with the chi-squared test, and continuous variables were compared with the unpaired Student's *t-*test and Mann-Whitney *U* test. Survival analysis was estimated by the Kaplan–Meier method and compared with the log-rank test. A Cox proportional hazards regression model was used in the multivariable analysis to identify the risk factors for recurrence and progression. Statistical analysis was performed using JMP software (Pro.13; SAS Institute, Cary, NC, USA). *P*-values were two-sided, and statistical significance was defined as *P* < 0.05 in all tests.

## SUPPLEMENTARY MATERIALS FIGURES


