# Supplementary material for: Extracellular miR-224 as a prognostic marker for clear cell renal cell carcinoma
Source: Oncotarget. 2017 Nov 15;8(66):109877–88. doi: 10.18632/oncotarget.22436 (PMC5746350; doi:10.18632/oncotarget.22436)
Supplement: Supplementary file 1 [file oncotarget-08-109877-s001.pdf]

Extracellular miR-224 as a prognostic marker for clear cell renal cell carcinoma

SUPPLEMENTARY MATERIALS

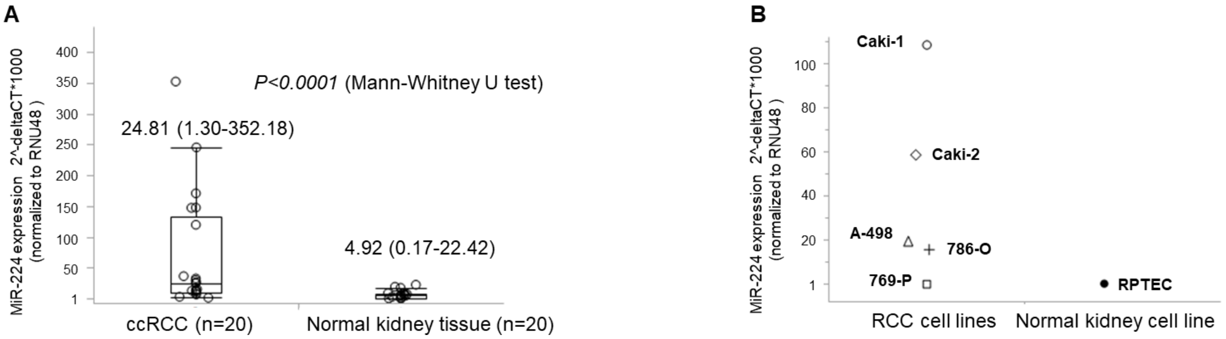

Supplementary Figure 1: Intracellular expression of miR-224 in ccRCC, matched normal kidney tissues, and cell lines. (**A**) miR-224 expression in ccRCC tissues (*n* = 20) and matched normal kidney tissues (*n* = 20). miR-224 expression was significantly higher in ccRCC tissues compared with matched normal tissues. (**B**) miR-224 expression in renal cancer cell lines (Caki-1, Caki-2, A-498, 786-O, and 769-P) and a normal renal cell line (RPTEC). The expression of miR-224 was higher in Caki-1 and Caki-2 cells compared with other renal cancer cell lines, while RPTEC had the lowest expression of miR-224.

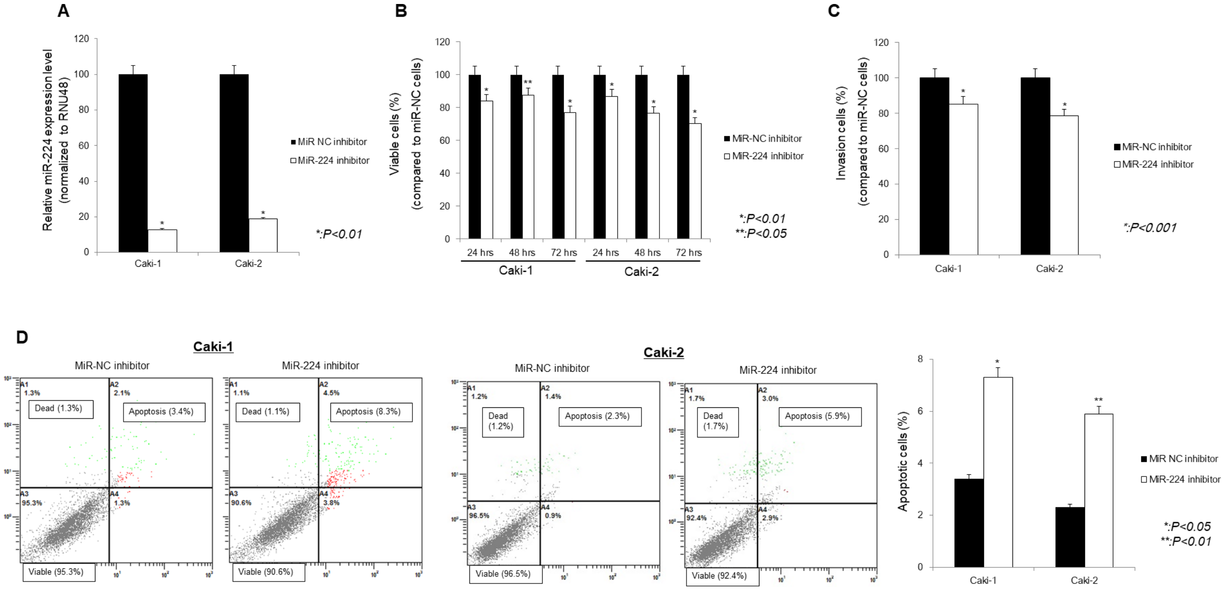

Supplementary Figure 2: Effect of downregulation of miR-224 on Caki-1 and Caki-2 cells. Cells transfected with an miR-224 inhibitor significantly decreased miR-224 expression compared with cells transfected with control (**A**). After miR-224 downregulation, cell growth (**B**) and cell invasion ability (**C**) were decreased while cell apoptosis was increased (**D**).

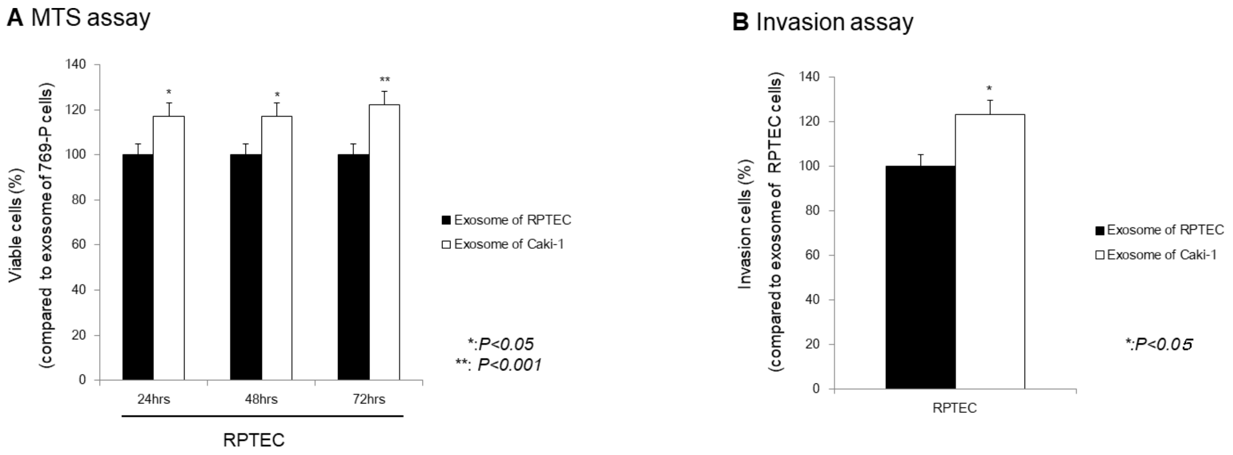

Supplementary Figure 3: Effect of addition of Caki-1 exosomes to target RPTEC cells using the MTS assay (**A**), Cell Invasion assay (**B**). (A, B) Caki-1 exosomes added to RPTEC cells significantly promoted cell viability and cell invasion compared with 769-P exosomes added to 769-P cells.
